# Adapting a Foundation Monocular Depth Model for Soccer Video: From Synthetic Supervision to Match-Level Reliability

**DOI:** 10.3390/s26134192

**Published:** 2026-07-02

**Authors:** Ju-Seong Do, Ho-Young Jung

**Affiliations:** Department of Artificial Intelligence, Kyungpook National University, 80 Daehak-ro, Buk-gu, Daegu 41566, Republic of Korea; jsdo@knu.ac.kr

**Keywords:** monocular depth estimation, sports computer vision, foundation model adaptation, soccer-video analysis, match-level reliability, computational efficiency, Depth Anything V2

## Abstract

Soccer-video analysis centers on pitch-plane tracking, but camera-view depth cues such as occlusion and goal-area structure are not fully represented on the field plane. Synthetic benchmarks provide dense supervision unavailable for real broadcasts, but whether adaptation yields predictions that are reproducible across matches and operationally feasible remains unclear. We evaluate a Depth Anything V2 model adapted to SoccerNet-Depth with four components: Unaligned MDE accuracy, scale-and-shift aligned diagnostic, match-to-match reliability, and accuracy–cost trade-off. The model achieves an unaligned validation AbsRel of 0.00372. The aligned diagnostic shows that Base DAv2 retained substantial scene-depth structure, whereas SoccerNet adaptation enabled direct compatibility with the normalized target without per-frame ground-truth fitting. Relative to the VKITTI-fine-tuned reference, the adaptation improved all eight metrics in all 21 validation matches, with paired Wilcoxon tests significant after Bonferroni correction. On the challenge split, it reduced AbsRel by 34.1% versus the official baseline. The higher-resolution configuration improved the validation AbsRel by 5.9%, while the default retained a better accuracy–cost balance. At 401.57 ms per frame, the default is suited to post-match analysis, not live or near-real-time use. The study contributes a benchmark-scoped adaptation case study and protocol for foundation MDE on SoccerNet-Depth.

## 1. Introduction

Modern soccer analysis is increasingly video-native. Player tracking, event detection, and tactical review are commonly organized around pitch-plane coordinates, but several visually important relations in broadcast footage are not fully represented on the field plane. Occlusion relationships, foreground–background separation, player overlap near the goal area, and the apparent depth of goal structures are camera-view properties. They influence how a single broadcast frame is interpreted, yet they are not directly recovered by GPS, local positioning systems, optical tracking, or homography-based pitch-plane localization.

Recovering three-dimensional structure in professional soccer has traditionally required multi-camera or sensor-assisted infrastructure. FIFA semi-automated offside technology, for example, uses dedicated stadium cameras and ball-mounted sensors to support offside decisions [1]. Low-infrastructure single-broadcast-view alternatives have become an active research direction in soccer, including monocular player localization on the pitch plane [2] and three-dimensional scene understanding based on broadcast replays [3]. Monocular depth estimation (MDE) is a natural extension of this trend because it predicts a dense camera-view depth field from a single image, potentially adding occlusion relationships and local scene structure to single-view sports-video analysis.

Recent foundation MDE models, including Depth Anything V2 (DAv2), have learned transferable depth priors from large-scale image datasets [4]. These models make domain adaptation more practical than training a soccer-specific depth model from scratch. Soccer video, however, is not a generic natural-image domain. It contains repeated player-scale actors, structured field-plane perspective, line markings, goal structures, broadcast camera motion, and large smooth pitch regions interrupted by sharp player and object boundaries. These regularities make foundation-model adaptation plausible, but they also make generic frame-averaged evaluation insufficient for sports-video deployment.

A key challenge in soccer-video depth evaluation is the absence of dense ground-truth depth for real broadcast footage. SoccerNet-Depth addresses this limitation by providing generated soccer-video frames with corresponding synthetic depth maps [5]. This setting does not by itself establish real-broadcast metric-depth validity. It does, however, provide a controlled intermediate benchmark in which target compatibility, match-level reproducibility, and workflow feasibility can be tested before claims are extended to real broadcast footage.

Standard MDE reporting usually averages pixel-wise errors over all frames. This is useful for benchmark comparison, but it is incomplete for soccer-video use. In SoccerNet-Depth, frames are grouped by match, so a frame-level average can hide failures that occur in particular matches. In addition, a prediction that becomes accurate only after fitting scale-and-shift to the ground truth is not the prediction that would be available at inference time. We also need to know whether errors from foundation MDE references arise only from scale-and-shift mismatch or whether they reflect incorrect soccer-scene depth structure. Finally, higher input resolution may slightly improve accuracy but increase latency, memory, and training cost beyond what a sports-video workflow can use.

For these reasons, this study evaluates an adapted DAv2 foundation MDE model using the four components unaligned mde accuracy, scale-and-shift aligned diagnostic, match-to-match reliability, and accuracy–cost trade-off. Together, these components test whether soccer-specific adaptation produces predictions that are accurate without ground-truth alignment, reproducible across matches, and practical for post-match soccer-video workflows.

Our adaptation case study, based on a DAv2 model initialized from a Virtual KITTI 2 synthetic-driving depth prior, produced four findings on SoccerNet-Depth. First, the adapted model produced unaligned predictions that were directly compatible with the normalized benchmark target. Second, the aligned diagnostic showed that the Base DAv2 relative prior retained substantial scene-depth structure under permissive per-frame affine alignment, whereas SoccerNet-specific adaptation enabled target-compatible output without ground-truth fitting. Third, the SoccerNet-specific adaptation gain over the VKITTI reference was reproduced across validation matches under paired non-parametric testing with multiple-comparison correction and leave-one-match-out sensitivity. Fourth, the accuracy–cost trade-off placed the tested configurations in the post-match processing tier, and the higher-resolution variant provided only a modest validation gain at substantially higher operational cost.

The contributions of this work are as follows.

- We adapt a DAv2 foundation MDE model to the SoccerNet-Depth soccer-video domain and evaluate, without ground-truth-based scale-and-shift alignment, whether its unaligned outputs are compatible with the benchmark target. We also separate the roles of the DAv2 foundation prior, the VKITTI initialization, and SoccerNet-specific adaptation using an ablation.
- We test whether the adaptation advantage is reproduced at the match level, treating each match as both the inferential unit and the natural unit of deployment in sports-video analysis.
- We quantify the accuracy–cost trade-off between the default and higher-resolution configurations and relate the result to post-match, near-real-time, and live sports-video workflows.

## 2. Related Work

### 2.1. Foundation Monocular Depth Estimation and Domain-Specific Adaptation

Deep learning-based monocular depth estimation (MDE) originated with multi-scale regression architectures for single-image depth prediction [6]. Robust multi-dataset training strategies, exemplified by MiDaS, established that a single model can transfer across diverse depth datasets under a relative-depth objective [7]. Transformer-based dense prediction further shaped the foundation MDE lineage [8]. The Depth Anything family, including DAv2, further scaled this direction through large-scale unlabeled data and teacher–student training, producing foundation-scale relative-depth predictors [4,9]. Image-based foundation MDE models do not inherently guarantee temporal consistency when applied frame by frame. Recent video-depth extensions of DAv2 introduce spatiotemporal modeling and temporal-gradient constraints to reduce frame-to-frame variation [10].

A standing distinction in the MDE literature separates relative-depth and metric-depth evaluation [11,12]. Relative-depth targets evaluate consistency with a normalized scene-depth representation rather than metric physical distance. SoccerNet-Depth follows the relative-depth convention with a normalized [0, 1] target [5]. The present study therefore treats evaluation on SoccerNet-Depth as benchmark-specific camera-view depth estimation, not as direct physical-distance measurement in real broadcast footage.

Domain-specific adaptation has become increasingly important as foundation depth models are applied beyond common indoor [13] and driving [14] benchmarks. Underwater MDE work has shown that terrestrial-trained foundation models fail under light attenuation and color distortion and that domain-specific fine-tuning is required for reliable performance [15]. Forest canopy work has adapted DAv2 for canopy-height estimation in remote sensing imagery with substantially reduced compute cost relative to domain-specific architectures [16]. In endoscopic surgery, parameter-efficient adaptation of vision foundation models is now standard, since zero-shot foundation models are not sufficient for surgical depth estimation [17]. Soccer video presents the same need for domain-specific adaptation. The additional property that frames are grouped by match has not been fully exploited in reliability analysis.

### 2.2. Soccer Vision, SoccerNet-Depth, and the SoccerNet 2025 Challenge

Artificial intelligence has been applied across many facets of football analysis, spanning statistical learning, game theory, and computer vision [18]. The same survey frames football computer vision as a source of spatially detailed representations, including depth estimation, for downstream analysis. Within computer vision specifically, the SoccerNet ecosystem provides large-scale benchmarks for soccer-video understanding, including action spotting [19], holistic broadcast-soccer understanding [20], multi-object tracking [21], game-state reconstruction [22], and broadcast-replay three-dimensional scene understanding [3]. Camera calibration and broadcast camera tracking on soccer video have been studied as separate tasks, with recent protocols arguing that planar field registration alone misses non-planar three-dimensional scene structure required for downstream applications [23,24]. SoccerNet-Depth complements this ecosystem by providing a sports-video MDE benchmark generated from a football video-game engine, with both RGB inputs and ground-truth depth maps being synthetic [5]. Synthetic supervision from video-game engines has broader precedent in computer vision [25]. Earlier soccer-specific work estimated player depth from a single broadcast frame using synthetic 3D player data extracted from football video games and reconstructed moving 3D scenes from real online soccer footage [26], providing an early precedent for synthetic supervision in monocular soccer reconstruction. The 2025 SoccerNet Monocular Depth Estimation Challenge reported top entries built on the Depth Anything V2 foundation model with various domain-adaptation strategies, such as full-resolution training with scale-and-shift-invariant and gradient-matching losses, player-focused correction networks, and segmentation-guided refinement, indicating that broadcast-soccer depth adaptation is an active direction [27]. The shared reliance on a Depth Anything backbone is consistent with the adaptation approach taken here, while the present study targets compatibility with the benchmark, match-level reliability, and computational cost rather than leaderboard-score optimization. Downstream applications of broadcast depth and three-dimensional scene structure include virtual banner replacement [28] and skeleton-oriented expected-goal estimation [29]. Accordingly, this work addresses the evaluation gap that remains after aggregate benchmark performance has improved, asking whether an adapted foundation MDE model produces unaligned outputs compatible with the SoccerNet-Depth target, whether scale-and-shift alignment removes the advantage, whether the advantage is reproduced across matches, and whether the configuration is feasible for sports-video workflows.

### 2.3. Reliability Evaluation with Match-Grouped Validation Frames

Sports and biomechanics evaluation distinguishes validity from reliability when assessing a system [30,31]. Validity refers to agreement with a reference, and reliability refers to stable performance across repeated conditions. Optical and broadcast-video tracking systems are routinely validated against motion-capture or multi-camera references with paired error reporting at the kinematic-variable level [32,33]. These conventions evaluate a system’s output against a reference, and they recognize that a system can have low average error while remaining unreliable under particular repeated conditions.

Standard AI benchmark reporting does not yet adopt this reliability-oriented evaluation framing. Frame-averaged pixel-wise metrics dominate, while paired reporting, per-group aggregation, and effect-size statements remain non-standard. Recent benchmark-evaluation work argues that point estimates from few repeated units under-represent uncertainty and recommends interval estimates with paired reporting and per-group aggregation [34]. Methodological work in sports analytics similarly emphasizes that evaluation choices must reflect the structure and intended use of the sporting data [35]. This concern is directly relevant to SoccerNet-Depth because validation frames are match-grouped rather than independently sampled, so frames within a match share the same rendering pipeline, lighting, camera trajectory, and stadium geometry. The match is therefore both a statistical group and a deployment episode for sports-video analysis. The present study transfers this reliability-oriented evaluation framing to an adapted soccer-video MDE pipeline by treating the match as the inferential unit and using paired non-parametric and sensitivity-based reporting.

### 2.4. Accuracy–Cost Trade-Offs in Domain-Adapted Deep Learning

Accuracy is often reported separately from operational cost in vision benchmarks, with training cost in particular under-reported alongside accuracy. For deployed domain-adapted MDE systems, however, accuracy alone is insufficient. Recent MDE/MMDE survey work also identifies computational cost and real-time deployment constraints as central practical issues for depth-estimation systems [36]. Underwater MDE benchmarking similarly evaluates foundation depth models across model scales and treats latency and memory demand as deployment-limiting factors in hardware-constrained settings [15]. Forest-canopy MDE adaptation has delivered task-relevant accuracy at substantially reduced compute cost relative to domain-specific architectures [16], and surgical depth estimation routinely uses parameter-efficient adaptation because surgical workflows tolerate only narrow inference budgets [17]. Cost-aware adaptation is therefore a recurring requirement when foundation MDE models move from benchmark comparison toward application workflows, not a constraint specific to sports.

Sports-video analysis imposes distinct workflow requirements. Live broadcast tactical analysis has been demonstrated at approximately 34–44 frames per second on a single RTX 3090 GPU in a real-time soccer analytics pipeline [37]. Near-real-time tactical overlay tolerates short-latency batched processing, and post-match review accommodates higher-cost analysis when accuracy gains justify it. Latency, peak GPU memory, and training-time requirements together determine whether a model is practical for post-match, near-real-time, or live sports-video use, even when its frame-averaged benchmark accuracy looks adequate. The present study therefore treats inference latency, peak VRAM, and training time as part of the evaluation protocol rather than as implementation details reported after accuracy. The accuracy–cost component is used to decide whether a higher-resolution configuration is a better default configuration or only a targeted re-analysis option.

## 3. Materials and Methods

The evaluation uses SoccerNet-Depth as the benchmark reference and follows the four-component protocol summarized in Table 1.

### 3.1. Experimental Setting

#### 3.1.1. Dataset and Benchmark Setting

We use SoccerNet-Depth [5] for all experiments. SoccerNet-Depth is generated from a football video-game engine, with depth maps extracted from the rendering pipeline. Both the input frames and the ground-truth depth maps are therefore synthetic. Each game in the SoccerNet-Depth release corresponds to one match, and we use the term match for the sports-domain unit throughout.

The dataset is divided into train, validation, and challenge splits, summarized in Table 2. We use the train split for model adaptation, the validation split for protocol diagnostics, configuration comparisons, and match-to-match reliability analyses, and the challenge split for official challenge test evaluation with ground truth withheld from participants.

The SoccerNet-Depth targets are normalized to [0, 1], and the reported metrics compare predictions with this benchmark-specific normalized target. In the released SoccerNet-Depth target used in this study, the normalized target follows an inverse-depth convention so that larger normalized values correspond to closer camera-view regions and smaller values correspond to farther camera-view regions. We use frames as the unit for frame-level depth metric computation and matches as the unit for reliability-focused aggregation.

#### 3.1.2. Model Adaptation and Training

**Figure 1:**
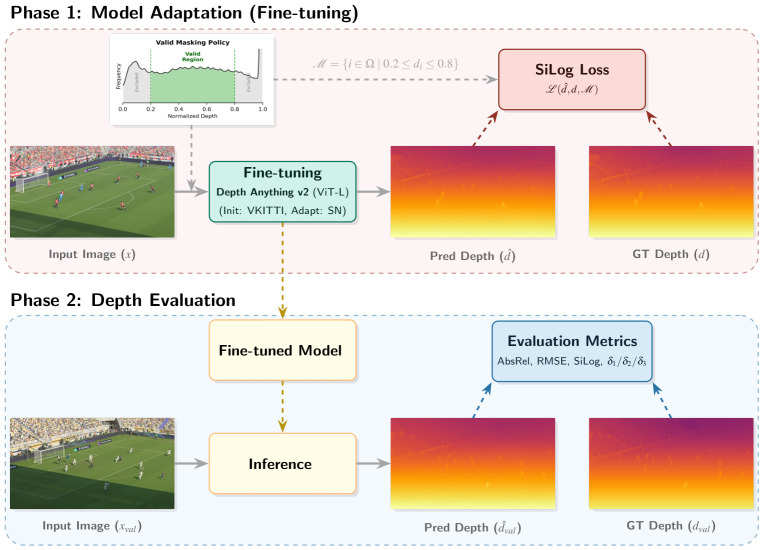
Model adaptation and depth evaluation pipeline. Phase 1 starts from the VKITTI-fine-tuned reference and adapts the Depth Anything V2 (DAv2) foundation MDE model on the SoccerNet-Depth train split using SiLog loss over the valid-pixel set M. Phase 2 performs inference on the validation split. Arrows indicate the pipeline flow from model adaptation to evaluation, and colored boxes distinguish data, model/training, prediction, ground truth, and metric components.

We adapt Depth Anything V2 (DAv2) with a ViT-L backbone as the foundation MDE model. We initialize the model from a DAv2 checkpoint fine-tuned on Virtual KITTI 2 [4,38], hereafter the VKITTI-fine-tuned reference, and then fine-tune it on the SoccerNet-Depth train split, following the pipeline in Figure 1, for 200 epochs using PyTorch version 2.5.1 with CUDA 12.1 and DistributedDataParallel on 4 × NVIDIA A40 GPUs. The 518 px configuration with batch size 4 per GPU is used as the default configuration and is referred to as Ours Default throughout the paper. A higher-resolution 672 px variant with batch size 2 per GPU is retained only for the accuracy–cost trade-off analysis. We optimize with AdamW under a polynomial learning-rate decay schedule. The exact values used for fine-tuning are provided in Appendix A (Table A1), and settings not stated there follow the Depth Anything V2 fine-tuning protocol [4].

Training uses the scale-invariant logarithmic (SiLog) loss. For predicted depth $\hat{d}$ and ground-truth depth *d* over a valid-pixel set M, the loss is

(1) $$L_{\mathrm{SiLog}}=\sqrt{\frac{1}{\left|M\right|}\sum_{i\in M}g_{i}^{2}-\frac{\lambda }{{\left|M\right|}^{2}}{\left(\sum_{i\in M}g_{i}\right)}^{2}},\;g_{i}=\log{\hat{d}}_{i}-\log d_{i},\;\lambda =0.5.$$

The unscaled SiLog evaluation metric uses the same expression, so the training objective and reported SiLog metric quantify the same scale-invariant log-error form.

The log-domain objective reduces sensitivity to proportional depth errors and is consistent with evaluating normalized SoccerNet-Depth targets. The valid-pixel set is defined as

(2) $$M=\{i\in \Omega |0.2\leq d_{i}\leq 0.8\}.$$

The valid-pixel protocol defines the pixels used for loss computation and metric evaluation. It does not modify the input image or the inference procedure. The band [0.2, 0.8] avoids extreme values, where log-based and ratio-based metrics become unstable. The same band is applied to all compared models. Across the 1441 validation frames, the band retained on average 97.3% of image pixels per frame.

For reference comparison, we evaluate two foundation MDE references without SoccerNet-specific fine-tuning. Base DAv2 represents the unadapted foundation relative-depth prior. The VKITTI-fine-tuned reference represents a synthetic-driving depth prior before SoccerNet-specific adaptation. Ours Default uses the same VKITTI-fine-tuned checkpoint after SoccerNet supervision, isolating the effect of soccer-specific adaptation on unaligned MDE accuracy.

### 3.2. Evaluation Protocol

#### 3.2.1. Unaligned MDE Accuracy

Unaligned MDE accuracy, the first deployment-realistic component, evaluates the model’s prediction directly, without ground-truth-based scale-and-shift alignment. Each estimator is evaluated in the form in which its prediction would be consumed at inference time. The pipeline includes the fixed model-specific inference and any ground-truth-independent resizing, but excludes any transformation estimated from validation ground truth. Following recent MDE evaluation work on alignment-dependent metric interpretation [39], no per-frame min–max normalization, percentile alignment, scale fitting, shift fitting, affine correction, or target-derived orientation correction is allowed.

All subsequent match-to-match reliability, stratified target-compatibility, and accuracy–cost analyses use this unaligned evaluation unless explicitly stated otherwise.

For predicted depth $\hat{d}$ and ground-truth depth *d* over the valid-pixel set M, the primary error metric is

(3) $$\mathrm{AbsRel}=\frac{1}{\left|M\right|}\sum_{i\in M}\frac{|{\hat{d}}_{i}-d_{i}|}{d_{i}}.$$

Following standard depth-estimation conventions, we additionally report SqRel, RMSE, and RMSElog. SqRel uses the squared error normalized by $d_{i}$, RMSE is the root mean squared depth error, and RMSElog is the same root mean squared error computed after applying the logarithm to ${\hat{d}}_{i}$ and $d_{i}$. The threshold metrics $\delta_{k}$ for k∈{1,2,3} are defined as the proportion of valid pixels with $\max\left({\hat{d}}_{i}/d_{i},d_{i}/{\hat{d}}_{i}\right)<1.25^{k}$. Lower values are better for the four error metrics. Higher values are better for the threshold metrics.

AbsRel is used as the primary interpretive metric in the validation and configuration analyses. All reported metrics are used for reliability analysis. The official SoccerNet challenge ranking uses RMSE as its primary criterion, and the official challenge test results therefore report the full official metric set.

We additionally partition the validation pixels by ground-truth depth structure to test whether the unaligned advantage is preserved in frame-level challenging regions.

The first factor partitions valid pixels by ground-truth depth range. The valid-pixel range [0.2, 0.8] is divided into three equal-width sub-ranges. For each frame, valid pixels are grouped into far ($d_{f,i}\in \left[0.2,\;0.4\right)$), mid ([0.4, 0.6)), and near ([0.6, 0.8]) ranges.

The second factor partitions valid pixels by ground-truth depth-gradient magnitude, computed on the full normalized depth map using finite differences. Within each frame, valid pixels at or above the 75th percentile of gradient magnitude form the boundary range, and the rest form the smooth range. This frame-adaptive threshold separates sharp depth discontinuities, such as player edges, occlusion seams, and goal-structure boundaries, from the smoother pitch-plane region.

For each range, depth metrics are computed using the pixels in that range without ground-truth fitting and aggregated to the match level for matches with at least one valid pixel in the range. Match-level paired Wilcoxon tests are then applied between Ours Default and the VKITTI-fine-tuned reference within each range, following the match-to-match reliability protocol. For the primary AbsRel comparison, Bonferroni correction is applied across the near, mid, far, boundary, and smooth ranges.

#### 3.2.2. Scale-and-Shift Aligned Diagnostic

The Scale-and-shift aligned diagnostic, hereafter the aligned diagnostic, tests whether the unaligned accuracy gap is recoverable by fitting a per-frame scale-and-shift to the validation ground truth. It complements the unaligned MDE accuracy by decomposing the unaligned gap into a component recoverable through affine alignment and a residual non-affine depth-structure component.

This diagnostic is intentionally favorable to the foundation MDE references. A separate ground-truth-fitted scale-and-shift are estimated for each frame using the validation ground truth. Therefore, if a foundation MDE reference remains far from the adapted model after this alignment, the residual gap cannot be attributed to a single global scale or offset mismatch.

For each validation frame *f*, we compute a per-frame scale coefficient $a_{f}$ and shift coefficient $b_{f}$. Following the scale-and-shift-invariant alignment convention used in relative-depth MDE evaluation [7,8], the pair minimizes the squared error against the ground-truth target over the valid-pixel set $M_{f}$ defined by Equation (2) in the SoccerNet-Depth normalized target domain:

(4) $$\left(a_{f},b_{f}\right)=\arg\min_{a,b}\sum_{i\in M_{f}}{\left(a{\hat{d}}_{f,i}+b-d_{f,i}\right)}^{2}.$$

The aligned prediction ${\tilde{d}}_{f,i}=a_{f}{\hat{d}}_{f,i}+b_{f}$ is then evaluated using the same metrics as the unaligned prediction.

This aligned score is a diagnostic quantity rather than a deployment metric. It measures how much of the unaligned gap can be removed by a ground-truth-fitted per-frame affine transformation, including possible orientation reversal, and how much remains as residual non-affine depth-structure disagreement. A large reduction after alignment indicates that the model preserves substantial scene-depth structure but uses an output convention that is incompatible with the SoccerNet-Depth target. The fitted correction is not available at inference time because it requires the ground truth.

#### 3.2.3. Match-to-Match Reliability

Each validation match is treated as the inferential unit for reliability analysis.

For each match g∈{1,…,21} and each metric, we average frame-level values for Ours Default and a specified reference estimator to obtain match-level means ${\bar{s}}_{g}^{\mathrm{Ref}}$ and ${\bar{s}}_{g}^{\mathrm{Ours}}$, where the average is over the frames $F_{g}$ belonging to match *g*. Each match contributes one mean per model and metric, regardless of frame count. In the match-level analysis, the reference model Ref is the VKITTI-fine-tuned DAv2 checkpoint evaluated before SoccerNet-specific adaptation.

We then compute direction-normalized paired improvements, so that positive values always favor Ours Default. For lower-is-better error metrics, $\Delta_{g}={\bar{s}}_{g}^{\mathrm{Ref}}-{\bar{s}}_{g}^{\mathrm{Ours}}$. For higher-is-better threshold metrics, $\Delta_{g}={\bar{s}}_{g}^{\mathrm{Ours}}-{\bar{s}}_{g}^{\mathrm{Ref}}$. The reliability quantity of interest is the median of the 21 paired match-level improvements for each metric.

For each metric, we test the following paired match-level hypotheses:

(5) $$\begin{array}{c} H_{0}:\mathrm{median}(\Delta_{g})=0,\\ H_{1}:\mathrm{median}(\Delta_{g})\neq 0. \end{array}$$

We use two-sided Wilcoxon signed-rank tests because the number of paired match-level units is small (G=21) and normality of the paired differences is not assumed. This follows standard non-parametric paired-comparison recommendations for comparing two algorithms over repeated datasets [40]. In this study, each validation match is treated as one repeated evaluation unit. Eight metrics are tested in parallel: AbsRel, SqRel, RMSE, RMSElog, SiLog, $\delta_{1}$, $\delta_{2}$, and $\delta_{3}$. We therefore apply Bonferroni correction with family-wise α=0.05, giving a corrected threshold of α=0.05/8=0.00625.

Median improvements are reported with bootstrap 95% confidence intervals from 10,000 paired resamples over matches.

Sensitivity to any single match is assessed by leave-one-match-out analysis, repeating the paired test on each 20-match subset and checking whether Bonferroni-corrected significance is preserved. As an assumption-light robustness check for directional reproducibility, we also report an exact two-sided sign test for the win/tie/loss pattern across matches. The sign test ignores improvement magnitude and tests whether the direction of improvement is consistently positive.

#### 3.2.4. Accuracy–Cost Trade-Off

We compare the accuracy–cost trade-off of the 518 px and 672 px configurations. We report inference latency, peak VRAM, and training time as operational metrics. Inference latency is measured as per-frame inference time on validation images. Peak VRAM is the maximum allocated GPU memory observed during inference. Training time is the per-image training time during fine-tuning. All profiling results are reported under the same hardware, precision setting, and software stack used for training. Latency is compared with post-match, near-real-time, and live-broadcast workflow requirements.

## 4. Results

### 4.1. Unaligned MDE Accuracy

The unaligned rows of Table 3 evaluate the first protocol component. This component asks whether each estimator’s native output is compatible with the normalized SoccerNet-Depth target without any ground-truth-derived correction. The same unaligned criterion is applied to all models, but unaligned numerical errors are not directly comparable as target-scale accuracy when a model’s native output convention has no fixed correspondence to the SoccerNet-Depth target. Base DAv2 produces relative-depth output with no fixed affine correspondence to the normalized target, so its direct numerical error primarily reflects output-convention mismatch rather than scene-depth quality. The VKITTI reference produces metric-depth output whose scale does not directly correspond to the normalized inverse-depth target. Ours Default, in contrast, produces native output in the normalized SoccerNet-Depth target convention.

The adapted model therefore produces target-compatible predictions at inference time without ground-truth-based correction. The later match-to-match reliability, stratified target-compatibility, and accuracy–cost analyses all use this same unaligned inference output.

In the unaligned inference-time setting, Ours Default achieved an AbsRel/RMSE/SiLog of 0.00372/0.00290/0.49. The VKITTI reference remained far from the normalized target (0.6755/0.3965/99.73), indicating that the synthetic-driving prior alone did not provide SoccerNet target compatibility. Base DAv2 produced much larger native unaligned errors (604.41/357.56/435.28) because its relative-depth output has no fixed target-scale correspondence, but its aligned diagnostic AbsRel of 0.00423 shows that the foundation prior still retained scene-depth structure once ground-truth-fitted affine alignment was allowed. Together, these rows separate the effects of the DAv2 foundation prior, the VKITTI synthetic-driving prior, and SoccerNet-specific adaptation. The foundation prior preserves structure under diagnostic alignment, the VKITTI reference remains target-incompatible in native unaligned form, and SoccerNet adaptation makes the native output compatible with the benchmark target. Appendix B (Table A2) extends this validation-side comparison into an ablation that also includes the higher-resolution variant.

We additionally stratify the unaligned evaluation by challenging regions defined from ground-truth depth structure. Table 4 reports range-wise metrics across two factors: depth band and depth gradient.

Ours Default reduced AbsRel relative to the VKITTI reference in every range. Bonferroni-adjusted Wilcoxon tests on the primary AbsRel comparison reached $p_{\mathrm{adj}}<0.001$ across all five depth and gradient ranges combined. The largest AbsRel gap appeared in the far depth range. AbsRel divides absolute error by the ground-truth target value, so the same absolute error produces a larger AbsRel for small targets. The far-range gap should therefore be interpreted as sensitivity to the target scale rather than as a statement about metric distance.

**Figure 2:**
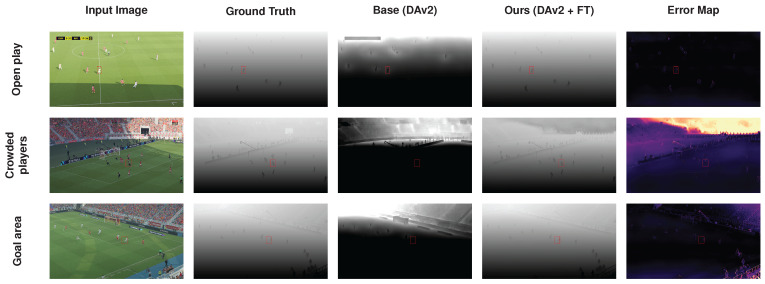
Qualitative comparison on three soccer-scene examples (open play, crowded players, goal area). Columns show RGB input, ground-truth depth, Base DAv2, Ours Default, and the Ours Default absolute error map. Depth panels use row-wise visualization normalization based on the ground-truth display range. The Base DAv2 panel is additionally orientation-matched and 90th-percentile-scaled for visual comparison only. The red boxes mark fixed reference regions for visual comparison across columns, and colors in the depth-map and error-map panels encode normalized depth or absolute-error magnitude for visualization.

**Figure A2:**
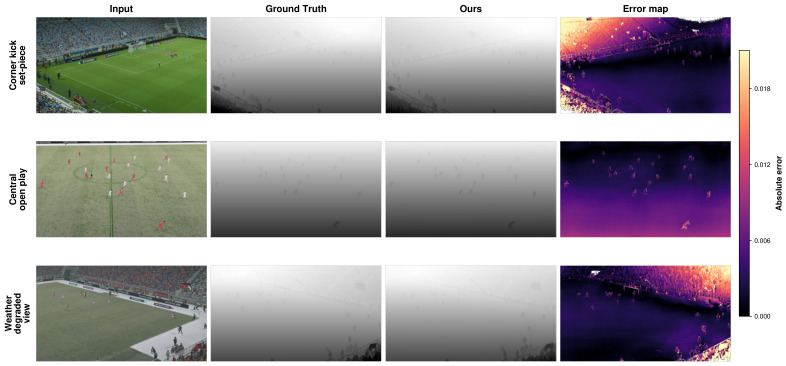
Challenging validation examples with elevated per-frame error for Ours Default. Each row shows the RGB input, ground-truth depth, the Ours Default prediction, and the absolute error map. The error maps use a shared display scale across the selected examples. The examples span a corner-kick set-piece (game 20, frame 72, AbsRel 0.0101), a central open-play scene (game 53, frame 26, AbsRel 0.0099), and a weather-degraded view (game 29, frame 4, AbsRel 0.0093). These selected per-frame values are elevated relative to the validation-set mean AbsRel of 0.00372 but remain near 0.01, showing higher error in visually difficult scenes without an unbounded increase.

The gap between the boundary and smooth ranges for Ours Default was negligible. Unaligned accuracy remained low for depth-discontinuity regions (player edges, occlusion seams, goal-structure boundaries) as well as for smooth pitch-plane regions. Figure 2 shows representative qualitative examples across all three scene types (open-play, crowded-player, goal-area), where Ours Default visually aligns with ground-truth depth while Base DAv2 shows pronounced depth-structure disagreement at player edges and goal-area boundaries. Representative challenging validation examples with elevated per-frame error, drawn from set-piece, open-play, and weather-degraded views, are provided in Appendix D (Figure A2).

**Figure 3:**
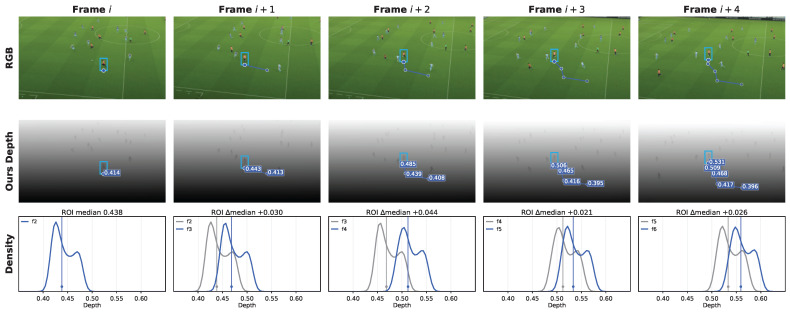
Qualitative depth behavior of Ours Default over a short continuous sequence from game 20, using frames 2–6 sampled at 1.2 fps (approximately 0.83 s between adjacent frames). The top row shows the RGB input with the tracked player region marked by a blue box, the middle row shows the predicted depth, and the bottom row shows the per-frame depth distribution within the tracked region. The median predicted depth within the tracked region changes smoothly across consecutive frames without an abrupt frame-to-frame jump. This sequence-level illustration is a qualitative diagnostic and not a dataset-wide temporal consistency metric.

Figure 3 provides a qualitative check of the unaligned Ours Default output over a short consecutive sequence, complementing the aggregate and stratified validation results. The same player region was tracked over five consecutive frames from one validation segment, and the median predicted depth within the region was computed in each frame. The predicted values changed smoothly across this example, without an abrupt frame-to-frame jump. This observation is qualitative and sequence-specific, and it does not constitute a dataset-wide temporal consistency metric.

### 4.2. Scale-and-Shift Aligned Diagnostic

**Figure 4:**
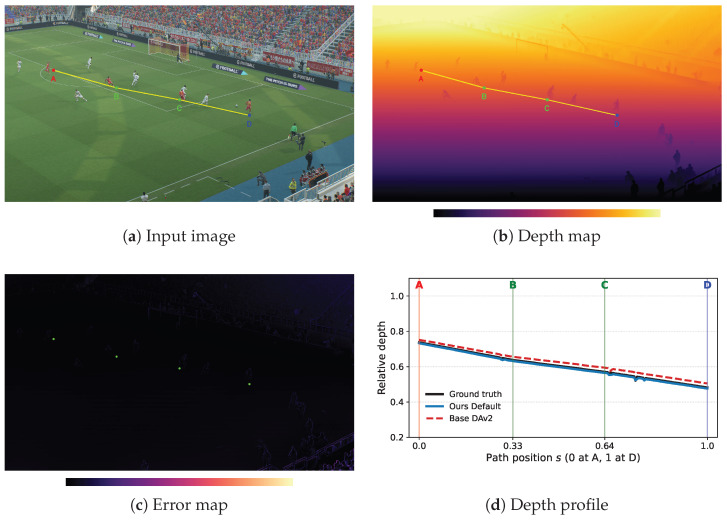
Camera-view depth profile along a tactical pitch trajectory on an example validation frame. (**a**) Input with reference points A, B, C, D. (**b**) Ours Default predicted depth. (**c**) Absolute error map against ground truth. (**d**) One-dimensional profile along A–D for ground truth (solid black), Ours Default (solid blue), and Base DAv2 (dashed red). The profiles are shown on a shared visualization scale using robust percentile normalization and orientation matching for visual comparison only.

Figure 4 provides a representative visual example in which Ours Default closely follows the local reference-depth variation along the trajectory on the visualization-normalized profile. The Base DAv2 curve is included only as a visual reference and is not part of the unaligned quantitative protocol.

The aligned rows of Table 3 provide the scale-and-shift aligned diagnostic. This diagnostic re-evaluates the same predictions after fitting an unconstrained per-frame scale-and-shift to the validation ground truth. For Base DAv2, the fitted scale is negative on all 1441 frames, so the aligned diagnostic also absorbs an orientation reversal relative to the SoccerNet-Depth inverse-depth convention. The diagnostic is therefore intentionally favorable to the foundation references by granting them a permissive per-frame affine correction that also permits orientation reversal. Appendix C presents the Base DAv2 evaluation diagnostic and explains why the main analysis adopts the native relative-depth output rather than the earlier metric-depth conversion.

Under this favorable alignment, the Base DAv2 error falls to the same order of magnitude as that of Ours Default, with AbsRel values of 0.00423 and 0.00141, respectively. This result indicates that the foundation relative prior already encodes substantial soccer-scene depth structure and that much of its direct target incompatibility arises from differences in scale, shift, and orientation rather than from an absence of scene-depth structure. The lower aligned error of Ours Default indicates an additional reduction in residual depth-structure error.

From a deployment perspective, Ours Default achieves an unaligned AbsRel of 0.00372, which is lower than the 0.00423 achieved by Base DAv2 only after per-frame ground-truth alignment. Although these values arise from different evaluation protocols, the comparison highlights that the adapted model produces target-compatible predictions without target-derived per-frame fitting. The principal benefit of SoccerNet-specific adaptation is therefore direct compatibility with the benchmark target without ground-truth-based alignment.

### 4.3. Match-to-Match Reliability

**Figure 5:**
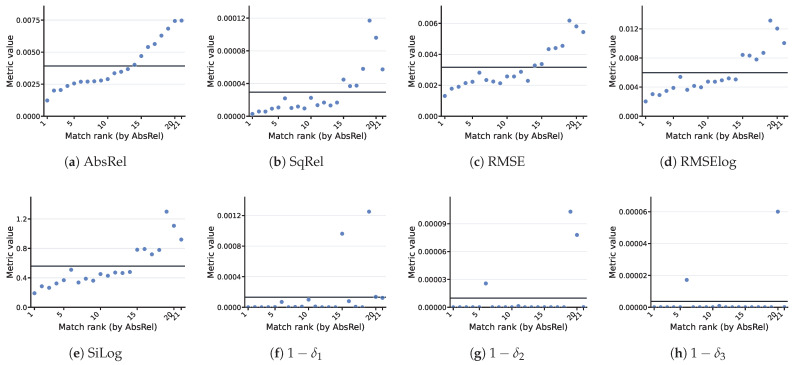
Per-match unaligned performance of Ours Default across the 21 validation matches. Each panel corresponds to one depth metric, and each point is the match-level mean for one match. Matches are ordered along the horizontal axis by AbsRel rank. Threshold metrics are shown as $1-\delta_{k}$, so that lower values indicate better performance, consistent with the error metrics. The horizontal line in each panel marks the across-match mean.

Figure 5 summarizes the per-match unaligned performance of Ours Default across the 21 validation matches. The error-metric panels show that performance remains low overall but still varies across matches. The $1-\delta_{k}$ panels remain close to zero, indicating that threshold-metric errors are rare.

**Figure 6:**
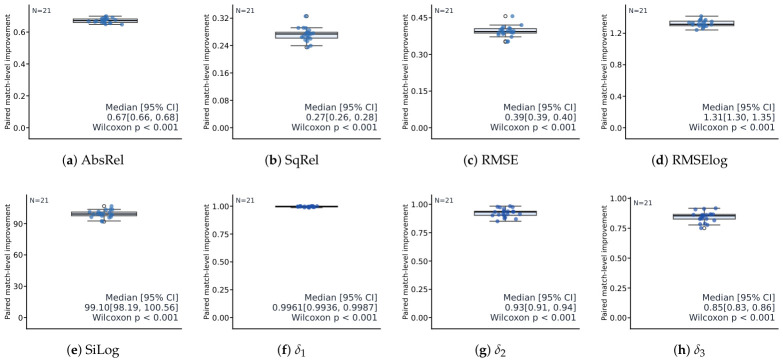
Paired match-level improvement of Ours Default over the VKITTI-fine-tuned reference across 21 validation matches. Each point is the direction-normalized difference between the VKITTI-fine-tuned DAv2 checkpoint before SoccerNet adaptation and Ours Default after SoccerNet adaptation. Positive values favor Ours Default. Each panel annotates median improvement, 95% CI, and two-sided Wilcoxon *p*.

To isolate the effect of SoccerNet-specific adaptation, Figure 6 compares Ours Default with the VKITTI-fine-tuned checkpoint used as its initialization. The two models share the same architecture and VKITTI-fine-tuned starting point and differ only in whether SoccerNet supervision is applied. Across all eight metrics, the direction-normalized paired differences are positive in all 21 matches, yielding a 21/0/0 win/tie/loss pattern. The median improvements and bootstrap 95% confidence intervals are positive in every panel.

The Wilcoxon signed-rank tests remain significant after Bonferroni correction (α = 0.00625). The exact two-sided sign test gives $p=9.54\times 10^{-7}$ for the 21/0/0 pattern, and $p_{\mathrm{adj}}=7.63\times 10^{-6}$ after Bonferroni correction across eight metrics. Leave-one-match-out sensitivity preserves Bonferroni-corrected significance for all eight metrics.

Together, these results show that the target-compatibility improvement introduced by SoccerNet-specific adaptation holds at the match level rather than only in pooled-frame averages. The 21 validation matches are not a single homogeneous condition. As summarized in Table 2, the train and validation splits cover comparable distributions of weather and time-of-day conditions. A simple metadata-balance check did not indicate a train–validation distribution difference for weather ($\chi^{2}$, p=0.9366) or time of day (Fisher’s exact test, p=0.7442).

Within the annotated condition diversity of the validation split, Ours Default improves over the VKITTI-fine-tuned reference in every validation match. This comparison isolates the contribution of SoccerNet supervision relative to the VKITTI-fine-tuned reference, and it does not establish superior reliability over other adapted SoccerNet-Depth submissions, for which per-match predictions are unavailable.

### 4.4. Accuracy–Cost Trade-Off

Table 5 compares the default 518 px configuration and the 672 px variant across model scale, compute, validation accuracy, and deployment cost.

The higher-resolution variant reduces validation AbsRel by 5.9%, while increasing FLOPs by 84.5%, inference latency by 60.0%, and peak VRAM by 91.4%. Because the architecture is unchanged, the parameter count is identical, whereas the per-frame FLOPs increase with input resolution. Under the measured hardware environment, both configurations operate within the post-match review tier rather than near-real-time or live-broadcast tiers. The 672 px configuration is therefore better interpreted as a targeted high-resolution option. The 518 px configuration remains the default for routine post-match processing.

### 4.5. Official Challenge Test Evaluation

Table 6 reports the official challenge split scores. The official baseline is a fine-tuned ZoeDepth model [27]. Because per-frame predictions from other challenge submissions are not publicly available, the comparison is interpreted as a score-level evaluation against the official baseline rather than as a controlled architectural comparison across submissions.

Relative to the official baseline, Ours Default reduces AbsRel by 34.1% and RMSE by 12.8%. The 672 px variant does not improve AbsRel over Ours Default and ties RMSE, although it is slightly lower on RMSElog and SiLog. Combined with the validation-side accuracy–cost trade-off, these scores support retaining the 518 px configuration as the default.

## 5. Discussion

Within the SoccerNet-Depth setting, the results support interpreting Ours Default as a benchmark-compatible depth estimator for soccer-video workflows. Its unaligned compatibility is not explained by per-frame scale-and-shift alignment alone. Under the aligned diagnostic, Base DAv2 becomes substantially closer to Ours Default, whereas the VKITTI reference remains worse after alignment. This pattern indicates that SoccerNet supervision improves target compatibility beyond a per-frame affine adjustment. The challenge result shows that the observation is not confined to the validation split, and the accuracy–cost analysis places the tested configurations in the post-match analysis regime.

These findings remain bounded by the SoccerNet-Depth setting. SoccerNet-Depth is a synthetic benchmark in which both the input frames and the depth targets are generated from the same rendering pipeline, so target compatibility on this benchmark does not by itself establish metric-scale physical-distance accuracy or external validity on real-broadcast footage. The official challenge test result reduces the concern that the reported advantage is specific to the 21 validation matches, but it does not rule out reliance on SoccerNet-Depth rendering regularities, since the challenge split belongs to the same synthetic benchmark family. Generalization to real broadcast video therefore remains an open question.

The reliability analysis is also limited by the match-level metadata available in the benchmark. Table 2 shows comparable weather and time-of-day distributions for the train and validation splits, but the released metadata does not annotate finer match-level production factors such as stadium identity, camera angle, lighting setup, and scene composition. We therefore cannot determine whether the splits differ in these factors or characterize how performance generalizes as they vary. We restrict the reliability claim to benchmark-level reproducibility across the evaluated validation matches rather than broader real-broadcast or deployment reliability.

The four-component protocol applies the same valid-pixel criterion uniformly across the full image, so valid non-field background regions are weighted together with the pitch, players, and goal structures. These background regions are peripheral to many player- and pitch-centered tactical applications, although they may remain relevant to camera calibration, broadcast compositing, and virtual-advertising tasks. Full-frame AbsRel should therefore not be read as a direct measure of downstream tactical utility. The present study does not compute semantic region-specific metrics. Separately evaluating the pitch, players, goal structures, and non-field background is left to future work.

Additional scope conditions apply within the protocol itself. The study evaluates single-frame depth and provides only a qualitative short-sequence temporal observation rather than a dataset-wide temporal consistency evaluation. The aligned diagnostic isolates only errors recoverable by affine alignment, and downstream tactical utility in coaching workflows is not evaluated.

## 6. Conclusions

This study evaluated an adapted Depth Anything V2 foundation MDE model on SoccerNet-Depth using a four-component sports-video protocol comprising unaligned MDE accuracy, scale-and-shift aligned diagnostic, match-to-match reliability, and accuracy–cost trade-off. The adapted model produced unaligned predictions that were directly compatible with the normalized benchmark target. The aligned diagnostic indicated that the foundation prior already retained substantial scene-depth structure and that SoccerNet-specific adaptation primarily improved target compatibility without per-frame ground-truth fitting. This target-compatibility improvement over the VKITTI-fine-tuned reference was reproduced across all 21 validation matches, the official challenge test split supported the validation-side finding, and the accuracy–cost analysis placed the tested configurations within a post-match processing regime rather than real-time use.

The contribution is a benchmark-scoped case study of adapting foundation MDE to soccer video using SoccerNet-Depth, supported by a four-component evaluation protocol. The study does not establish architectural superiority or real-broadcast metric-depth validity. Real-broadcast validation, temporally consistent video-depth modeling, cross-sport generalization, and cross-architecture comparison remain future work.

## Figures and Tables

**Table 1:** Four-component sports-video evaluation protocol for foundation monocular depth estimation on SoccerNet-Depth.

Component	Evaluation Question	Role in Sports-Video Use
Unaligned MDE accuracy	How accurate is the MDE output against the SoccerNet-Depth reference without ground-truth-based scale-and-shift alignment?	Downstream sports-video modules consume predictions without access to reference depth for post hoc alignment.
Scale-and-shift aligned diagnostic	How much error remains after per-frame scale-and-shift alignment to validation ground truth?	Separates output-convention mismatch from residual non-affine disagreement. Diagnostic only and not deployable.
Match-to-match reliability	Does the improvement hold across matches rather than only in pooled frames?	Tactical analysis is performed match by match, and failures within a subset of matches can be hidden by frame-level averages.
Accuracy–cost trade-off	Does higher resolution justify its latency, memory, and training cost?	Post-match review, near-real-time, and live workflows have different compute budgets.

**Table 2:** SoccerNet-Depth dataset overview. All frames use 1920 × 1080 resolution. Weather counts are reported as sunny/rainy/snowy match counts, and time-of-day counts are reported as day/night. Dashes indicate challenge-split weather and time-of-day metadata that are not publicly released.

	Data Statistics				Metadata	
Split	#Matches	#Frames	Frames/Match	GT	Weather	Day/Night
Train	21	4071	194	Yes	12/6/3	15/6
Validation	21	1441	69	Yes	13/5/3	13/8
Challenge	20	2615	131	No	—	—

**Table 3:** Unaligned MDE accuracy and the scale-and-shift aligned diagnostic on the SoccerNet-Depth validation split. All models are evaluated on all 1441 validation frames. Aligned rows use an unconstrained per-frame affine fit to validation ground truth and are diagnostic only.

Model	Evaluation	AbsRel	RMSE	SiLog
Base DAv2	Unaligned	604.41	357.56	435.28
	Aligned	0.00423	0.00363	0.68
VKITTI reference	Unaligned	0.6755	0.3965	99.73
	Aligned	0.0696	0.0445	8.24
Ours Default	Unaligned	0.00372	0.00290	0.49
	Aligned	0.00141	0.00179	0.31

**Table 4:** Depth- and gradient-stratified unaligned target compatibility before and after SoccerNet-specific adaptation on the SoccerNet-Depth validation split. The far depth band aggregates 18 of 21 matches because three matches contained no valid pixels there. Bonferroni-adjusted *p*-values are reported across the five depth and gradient ranges combined, with family-wise α=0.05. Dashes indicate values that are not applicable for reference rows.

Range	$N_{g}$	Method	AbsRel	RMSE	SiLog	AbsRel $p_{\mathrm{adj}}$
Depth range						
Near range	21	VKITTI reference	0.5622	0.3943	71.51	–
(0.6–0.8)		Ours Default	0.0028	0.0029	0.36	<0.001
Mid range	21	VKITTI reference	0.8007	0.4149	117.54	–
(0.4–0.6)		Ours Default	0.0038	0.0023	0.37	<0.001
Far range	18	VKITTI reference	0.8067	0.2955	116.91	–
(0.2–0.4)		Ours Default	0.0102	0.0045	1.07	<0.001
Depth-gradient range						
Boundary range	21	VKITTI reference	0.6914	0.4018	101.26	–
(≥75th pct)		Ours Default	0.0038	0.0030	0.51	<0.001
Smooth range	21	VKITTI reference	0.6374	0.3840	94.91	–
(<75th pct)		Ours Default	0.0039	0.0031	0.52	<0.001

**Table 5:** Accuracy–cost comparison between 518 px and 672 px configurations. FLOPs are measured for one forward pass per frame at inference. Downward arrows (↓) mark metrics for which lower values are better.

Metric	518 px	672 px	Change
Model and compute			
Parameters (M)	335.3	335.3	0.0%
FLOPs (G/frame) ↓	1298.7	2396.2	+84.5%
Accuracy			
Validation AbsRel ↓	0.00372	0.00350	−5.9%
Deployment cost			
Inference latency (ms/frame) ↓	401.57	642.61	+60.0%
Peak VRAM (GB) ↓	15.34	29.36	+91.4%
Training time (ms/image) ↓	257.9	359.9	+39.6%

**Table 6:** Official challenge split benchmark results.

Method	AbsRel	SqRel	RMSE	RMSElog	SiLog
Official challenge baseline	0.00402	0.00004	0.00376	0.00689	0.69
Ours Default	0.00265	0.00004	0.00328	0.00577	0.58
Ours 672 px	0.00268	0.00004	0.00328	0.00572	0.57

**Figure A1:**
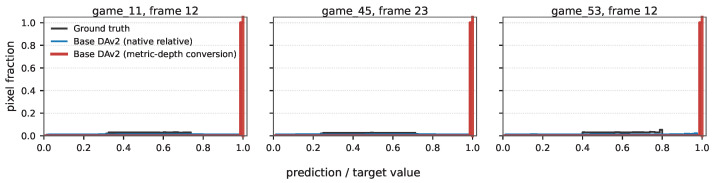
Per-frame pixel-value distributions on three representative validation frames. In each column, the ground-truth target (black) and the native relative-depth Base DAv2 output (blue) span the valid range, whereas the Base DAv2 output produced by the metric-depth conversion setting (red) is saturated near the upper bound. For the metric-depth conversion curve in the displayed examples, the valid-pixel distribution collapses to min=max=1.0 with std=0, yielding rank-deficient per-frame affine fits and explaining why alignment under this setting failed on a subset of frames.

## Data Availability

The SoccerNet-Depth dataset is publicly available through the SoccerNet data page at https://www.soccer-net.org/data (accessed on 28 June 2026) and through the SoccerNet/SN-Depth-2025 repository at https://huggingface.co/datasets/SoccerNet/SN-Depth-2025 (accessed on 28 June 2026). Challenge test ground truth is not publicly released. No primary data were collected for this study.

## Appendix A. Training Configuration

Table A1 summarizes the exact hyperparameter values used in our SoccerNet-Depth fine-tuning runs. Shared settings are listed once, and resolution-specific settings are separated for the 518 px default and 672 px variant.

**Table A1:** Training hyperparameters for SoccerNet-Depth adaptation of Depth Anything V2 (ViT-L).

Setting	Value
Shared fine-tuning settings	
Backbone	ViT-L (Depth Anything V2)
Initialization	VKITTI-fine-tuned DAv2
Optimizer	AdamW
Encoder learning rate	$5\times 10^{-6}$
Depth-head learning rate	$2.5\times 10^{-5}$
Learning-rate schedule	Polynomial decay, power 0.9
Warmup	None
Adam betas	(0.9, 0.999)
Weight decay	0.01
Epochs	200
Image normalization	ImageNet mean and standard deviation
Data augmentation	Horizontal flip, p=0.5
Loss	SiLog, λ=0.5
Valid-pixel band	[0.2,0.8]
Depth output range	[0.001,1.0]
Resolution-specific settings	
Resize/crop size	518×518 (default), 672×672 (high resolution)/random square crop
Batch size per GPU	4 for 518 px, 2 for 672 px (NVIDIA A40)

## Appendix B. Ablation

This appendix presents an ablation that adds one factor at a time to the foundation model, isolating the contribution of the VKITTI initialization, SoccerNet-specific adaptation, and higher input resolution in turn. All rows report unaligned native-output metrics on the validation split. The first two rows have no fixed correspondence to the SoccerNet-Depth target, so their values reflect target-scale mismatch rather than scene-depth quality, as established in the unaligned MDE accuracy analysis. The principal evidence for this contribution is also given by Table 3 and by the VKITTI-versus-Ours match-level and stratified results in Figure 6 and Table 4.

**Table A2:** Ablation adding one factor at a time on unaligned native output. Each row adds a single factor to the row above. The first two rows have no fixed correspondence to the SoccerNet-Depth target, so their values reflect target-scale mismatch rather than scene-depth quality.

Configuration	AbsRel	RMSE	SiLog
Base DAv2	604.41	357.56	435.28
+VKITTI initialization	0.6755	0.3965	99.73
+SoccerNet adaptation	0.00372	0.00290	0.49
+higher resolution (672 px)	0.00350	0.00273	0.50

## Appendix C. Base DAv2 Evaluation Diagnostic

This appendix documents the Base DAv2 evaluation diagnostic used to explain why the main analysis adopts the native relative-depth output rather than the earlier metric-depth conversion. It is not an alternative evaluation with equivalent standing to the main analysis. The diagnostic record is retained for transparency and explains the origin of the previously reported Base DAv2 aligned values.

The main analysis evaluates Base DAv2 on its native relative-depth output. An earlier diagnostic instead evaluated Base DAv2 through a metric-depth conversion, which interprets the foundation output on a fixed metric-depth scale. Native relative-depth Base DAv2 retains substantial structure but lacks direct target-scale compatibility. The earlier metric-depth conversion produced numerically smaller unaligned values but introduced saturation and incomplete affine alignment. The main analysis therefore adopts the native relative-depth output, while the earlier conversion results are retained only as a transparent diagnostic record.

Base DAv2 produces relative-depth output with no fixed affine correspondence to the SoccerNet-Depth target. Under the metric-depth conversion setting, the stored prediction was saturated near the upper bound on a subset of frames, so the per-frame valid-pixel values collapsed to a near-constant field. When the prediction is near-constant, the per-frame affine fit defined in the main text is rank-deficient and the scale-and-shift fit is degenerate rather than informative.

An alignment under the metric-depth conversion setting is treated as failed if the least-squares system is singular, if the aligned prediction contains non-positive or non-finite values over the valid pixels, or if the resulting values violate the domain of the log-based or ratio-based metrics. Under this setting, 484 of the 1441 validation frames met a failure criterion, and the aligned score in Table A3 is computed on the remaining 957 frames. The native relative evaluation used in the main text has no such failures and retains all 1441 frames.

**Table A3:** Diagnostic comparison of Base DAv2 under the earlier metric-depth conversion setting and the native relative-depth output adopted in the main analysis.

Evaluation Setting	Evaluation	Frames Used	AbsRel	RMSE	SiLog
Native relative-depth output	Unaligned ^a^	1441/1441	604.41	357.56	435.28
	Aligned	1441/1441	0.00423	0.00363	0.68
Metric-depth conversion	Unaligned	1441/1441	0.7504	0.4136	42.73
	Aligned	957/1441	0.2055	0.1313	23.21

^a^ The native relative-depth output has no fixed correspondence to the normalized SoccerNet-Depth target. Its unaligned values document output-convention mismatch rather than scene-depth quality. Aligned rows are diagnostic only, and the metric-depth conversion setting is retained only for transparency.

## Appendix D. Challenging Validation Examples with Elevated Error

Figure A2 shows validation frames for which Ours Default has elevated per-frame error, complementing the aggregate and stratified results in the main text. The examples are selected from the evaluated validation frames, which correspond to the main camera feed (video_1), and are intended to illustrate where error increases rather than to define an exhaustive failure taxonomy. They cover a corner-kick set-piece with dense-player context and goal-structure boundaries, a central open-play scene with many players, and a weather-degraded view. The released validation frames do not carry explicit replay-view or camera-cut annotations, so those view types are outside the scope of the evaluated set.
